# Low expression of long noncoding RNA CTC‐297N7.9 predicts poor prognosis in patients with hepatocellular carcinoma

**DOI:** 10.1002/cam4.2618

**Published:** 2019-11-01

**Authors:** Sicong Zhu, Xuelian Huang, Kelin Zhang, Wenliang Tan, Zhirong Lin, Qing He, Yajin Chen, Changzhen Shang

**Affiliations:** ^1^ Guangdong Provincial Key Laboratory of Malignant Tumor Epigenetics and Gene Regulation Sun Yat‐sen Memorial Hospital Sun Yat‐sen University Guangzhou Guangdong People's Republic of China; ^2^ Department of SICU Sun Yat‐sen Memorial Hospital Sun Yat‐sen University Guangzhou Guangdong People's Republic of China; ^3^ Department of Anesthesiology Sun Yat‐sen Memorial Hospital Sun Yat‐sen University Guangzhou Guangdong People's Republic of China; ^4^ Department of Hepatobiliary Surgery Sun Yat‐sen Memorial Hospital Sun Yat‐sen University Guangzhou Guangdong People's Republic of China

**Keywords:** biomarker, CTC‐297N7.9, hepatocellular carcinoma, long noncoding RNA, prognosis

## Abstract

**Background:**

Long noncoding RNAs (lncRNAs) are reported to play important roles in tumorigenesis of various malignant tumors. However, the clinical significance of aberrant lncRNA expression in hepatocellular carcinoma (HCC) is still elusive.

**Methods:**

Firstly, a differentially expressed lncRNA CTC‐297N7.9 in HCC was detected by analyzing the data from The Cancer Genome Atlas (TCGA). Secondly, the expression level of CTC‐297N7.9 was examined in four HCC cell lines and 60 pairs of HCC tissues by polymerase chain reaction (PCR) assay at our center. Thirdly, receiver operating characteristic (ROC) analysis was performed to evaluate the diagnostic value of CTC‐297N7.9 for HCC. Correlation and survival analysis of HCC patients from the TCGA and our center were also carried out to assess the predictive value of CTC‐297N7.9. Finally, survival prognostic models were established combining lncRNA expression and other clinical parameters.

**Results:**

The expression of CTC‐297N7.9 was downregulated in HCC cell lines and HCC tissues. ROC curve revealed its significant diagnostic value in HCC. CTC‐297N7.9 expression correlated with serum alpha‐fetal protein (AFP), tumor stage, and tumor differentiation. Survival analysis indicated that overall survival (OS) and disease‐free survival (DFS) are all positively associated with CTC‐297N7.9 expression, especially in patients without viral hepatitis or cirrhosis. Cox regression analysis showed that CTC‐297N7.9 expression level is an independent prognostic factor for both OS and DFS in HCC patients. Based on the model, CTC‐297N7.9 was observed to be negatively correlated to risk score, indicating its role as a protective factor for HCC.

**Conclusion:**

Our study demonstrated that the low expression of CTC‐297N7.9 is associated with poor prognosis in HCC patients, suggesting its possible role as a potential prognostic marker for HCC.

## Graphical abstract

Our study demonstrated a novel long noncoding RNA CTC‐297N7.9, which expression was negatively correlated with the prognosis of hepatocellular carcinoma (HCC) patients, using bioinformatics analysis method and tissue specimen. CTC‐297N7.9 was proved to have potential to be a prognostic biomarker for HCC.

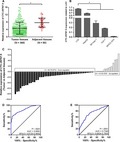

## 1 INTRODUCTION

Hepatocellular carcinoma (HCC) is the most prevalent malignant tumor with approximately 8.5 million new cases and 8.1 million deaths reported each year.^1^ Nowadays, great improvement has been made in the therapy of HCC thanks to the encouraging progress in early diagnosis and cancer therapeutic methods such as liver transplantation, surgical resection, local ablation, and transcatheter arterial chemoembolization.^2^, ^3^ However, the high invasion and metastasis rate of HCC have led to unfavorable prognosis in its patients.^4^ The overall survival (OS) rate of HCC patients remains dissatisfying, mainly due to HCC's insidious onset and its high rate of recurrence.^5^ Survival prognosis of HCC patients vary considerably due to various factors. There is a lack of sensitive and specific clinical indicators to predict the prognosis in HCC patients. Therefore, it is vital to look for an effective biomarker that is significant in directing the treatment of HCC.

Aberrant accumulation of genetic and epigenetic alterations often causes the initiation and development of cancer.^6^ Long noncoding RNAs (lncRNAs), a novel type of RNA molecules with sizes larger than 200 nucleotides in length but has limited capability in protein coding, have become a hot research topic and have been widely studied in antitumor‐related fields.^7^ Long noncoding RNAs were identified to function similarly to oncogene and tumor suppressor genes that are associated with the biological behavior of malignant tumors such as proliferation, differentiation, apoptosis, and metastasis.^8^, ^9^, ^10^ Several studies have also demonstrated that lncRNAs could serve not only as biomarkers for diagnosing a diversity of human cancers, but also as predictive factors for patient survival. For example, HOX Antisense Intergenic RNA (HOTAIR), a well‐characterized lncRNA, was reported to be a biomarker for multiple tumors including breast cancer, colorectal cancer, pancreas cancer, and liver cancer, which were associated with poor patient prognosis.^11^, ^12^, ^13^, ^14^, ^15^ Besides, lung adenocarcinoma transcript 1 (MALAT1), also known as NEAT2 for nuclear‐enriched abundant transcript 2, was also proven to act as an oncogene that participates in the progression of lung cancer, cervical cancer, gastric cancer, ovarian cancer as well as HCC, and has become a new target in antitumor therapy.^16^, ^17^ Recently, a great number of novel lncRNAs were found to play important roles in the development of HCC, yet the relationship between lncRNAs and HCC remains elusive.

In this study, we aim to find a novel lncRNA that can be used to predict the prognosis of HCC patients and might serve as a novel biomarker for HCC. After analyzing the lncRNA expression profiles from The Cancer Genome Atlas (TCGA) database through bioinformatics analysis, we focused on lncRNA CTC‐297N7.9, which was differentially expressed in HCC tissues at a significant level. Hepatocellular carcinoma cell lines and surgical specimens were used to further confirm the aberrant regulation in this carcinoma. Correlation and survival analysis were performed in the patients from the TCGA database and our center to investigate the possible role of CTC‐297N7.9 in HCC diagnosis and to evaluate its accuracy in predicting the outcomes of HCC patients. In our study, we identified that CTC‐297N7.9, which was downregulated in HCC, can serve as an indicator for poor prognosis in HCC patients.

## 2 MATERIALS AND METHODS

### 2.1 Cell lines and cell culture

Hepatocellular carcinoma cell lines, HepG2, SK‐hep‐1, Huh‐7, and the normal liver epithelium cell line LO2, were purchased from the Cell Bank of the Chinese Academy of Sciences. Meanwhile, another HCC cell line, MHCC‐97H, was purchased from Shanghai Zhong Qiao Xin Zhou Biotechnology Company. All cell lines were cultured in high‐glucose Dulbecco's modified eagle medium (Gibco) supplemented with 10% fetal bovine serum (Biological Industries), 100 U/mL penicillin, and 100 mg/mL streptomycin. All cells were maintained at 37.0°C in an atmosphere containing 5% CO_2_.

### 2.2 Patients and tissue samples

Hepatocellular carcinoma tissues and adjacent normal tissues were collected from 60 HCC patients who had undergone hepatectomy in Sun Yat‐sen Memorial Hospital between 2015 and 2017. Before surgery, none of the patients had received radiotherapy, chemotherapy, transcatheter arterial chemoembolization nor treated with molecular targeted drugs such as Sorafenib. All the patients were diagnosed with HCC by postoperation pathology. Adjacent tissues were obtained 2.0‐cm distal from tumor tissue. The resected specimens were immediately frozen in liquid nitrogen and stored at −80°C until RNA extraction. All the patients had undergone clinical follow‐up for more than 1 year. All patients were reexamined 1 month after operation, and once every 3 months after that. At each follow‐up visit, the patient's liver function and serum alpha‐fetal protein (AFP) level were checked, while imaging examinations such as ultrasound, CT, and MRI scan were also done in order to detect tumor recurrence. Besides, the information on survival and postoperative conditions of the patients was also obtained through monthly follow‐up telephone calls. This study has been approved by the Ethics Committee of Sun Yat‐sen Memorial Hospital, Sun Yat‐sen University. Informed consent form was signed by all participants who were involved in this study too.

### 2.3 RNA extraction and quantitative reverse transcription PCR

Total RNA was extracted from tissue specimens and cell lines by Trizol reagent (Takara). cDNA was synthesized with PrimeScript RT reagent Kit according to the manufacturer's instructions. Quantitative reverse transcription polymerase chain reaction (PCR) was performed on CFX96 system (BIO‐RAD) using SYBR Premix Ex Taq (Takara). The PCR conditions for all assays were set at 95°C for 30 seconds, followed by 40 cycles of amplification (95°C for 5 seconds and 60°C for 20 seconds). GAPDH (Glyceraldehyde 3‐phosphate dehydrogenase) was used as endogenous controls. The relative expression of CTC‐297N7.9 was calculated using the 2^−ΔΔCT^ method.^18^ The primers were provided as follows: CTC‐297N7.9 forward: 5′‐TCCATAGGTGATCACTGCGG‐3′, reverse: 5′‐CAGCAGCAGAAGGGGATTTG‐3′; GAPDH forward: 5′‐CCAGAACATCATCCCTGCCT‐3′, reverse: 5′‐CCTGCTTCACCACCTTCTTG‐3′. Expression levels were presented as fold change (FC) of corresponding controls which were defined as 1.0.

### 2.4 Data acquisition from the TCGA database

The gene expression data of HCC and clinical information of HCC patients were obtained from the Genomic Data Commons Data Portal within the TCGA data portal (https://portal.gdc.cancer.gov/) using R (http://r-project.org; software version 3.4.1). The clinical information of 377 HCC patients from the TCGA and gene expression profiles of 427 tissue samples, including 377 tumors and 50 adjacent tissues, were downloaded. Patients with crucial clinical prognosis data missing were eliminated. Finally, a total of 368 HCC patients with gene expression data of 368 tumors and 50 adjacent tissue samples were included in our study.

### 2.5 Analysis of differentially expressed lncRNAs

RNA‐sequencing data (Fragments Per Kilobase Million normalization, FPKM normalization) of 50 HCC and corresponding adjacent tissue samples were compared to identify lncRNAs with aberrant expression in tumor tissues. The FCs of lncRNA expression between tumor and adjacent tissues were calculated by *R*, and the *P* value was adjusted by multiple testing using the false discovery rate method. With |FC| > 2.0 and *P* < .05, the differentially expressed lncRNAs (DElncRNAs) in HCC were identified.

### 2.6 Identification of diagnostic and prognostic value of lncRNAs

In order to evaluate the diagnostic significance of DElncRNAs in HCC, receiver operating characteristic (ROC) analysis was performed. The standards for assessing the area under the ROC curve (AUC) were as follows: 0.50‐0.70 suggest poor evidence for diagnosis; 0.70‐0.85 indicate moderate evidence for diagnosis; while 0.85‐1.00 imply high evidence for diagnosis. Furthermore, survival analysis was conducted to identify the relationship between the expression level of lncRNAs and the outcome of HCC patients. The selected DElncRNAs were all analyzed by univariate survival analysis to evaluate their prognostic significance in HCC.

### 2.7 Construction of HCC risk score system

The selected lncRNAs that were obtained from ROC and univariate analysis were included in the Cox regression analysis. This analysis was carried out to construct a suitable model that is associated with patient survival using the least number of clinicopathological characteristics. The scores of clinical features were defined as either 1 or 0 based on differences in clinicopathological characteristics of HCC patients. The regression coefficients were calculated through multivariate regression analysis. Then, survival prognostic model made up of lncRNA and clinical characteristics was established, and a risk score was computed as follows: Risk score = $\sum_{i=1}^{N}C_{i}\times S_{i}$ (“*N*” is the number of prognostic factors, “*C*” denotes the regression coefficient obtained from the multivariate regression model, and “*S*” refers to the scores of clinical features). Using median risk score as the threshold, the HCC patients were stratified into high‐ and low‐risk groups. The difference in prognosis between high‐ and low‐risk groups was also compared to evaluate the efficacy of the survival prognostic models.

### 2.8 Statistical analysis

Differentially expressed lncRNA analysis was performed with DESeq2 package of R, and univariate survival analysis of DElncRNAs was done using Survival package of R. Charting and hierarchic cluster analyses of the heatmap were conducted by the ImageGP software (http://www.ehbio.com/ImageGP/index.php). Quantitative variables were evaluated by independent sample *t* test or ANOVA, as appropriate; qualitative variables were analyzed using Chi‐square test. Overall and disease‐free survival (DFS) curves were constructed using the Kaplan‐Meier (K‐M) methods and compared using a log rank test. Univariate and multivariate analyses were evaluated by Cox regression method, and the regression coefficient of survival prognostic model was also calculated using Cox analysis. Receiver operating characteristic analysis was utilized to assess the efficiency of HCC diagnosis and prognosis prediction. All the *t* test, ANOVA^2^ tests, Cox analysis, K‐M survival curves, and ROC curves were carried out with SPSS 22.0 software (IBM Corporation). All PCR assays were repeated for at least three times, and presented as mean ± SD. In our study, *P* values less than .05 were considered as statistically significant.

## 3 RESULTS

### 3.1 Identification of key lncRNAs relating to diagnosis and prognosis

**Figure 1:**
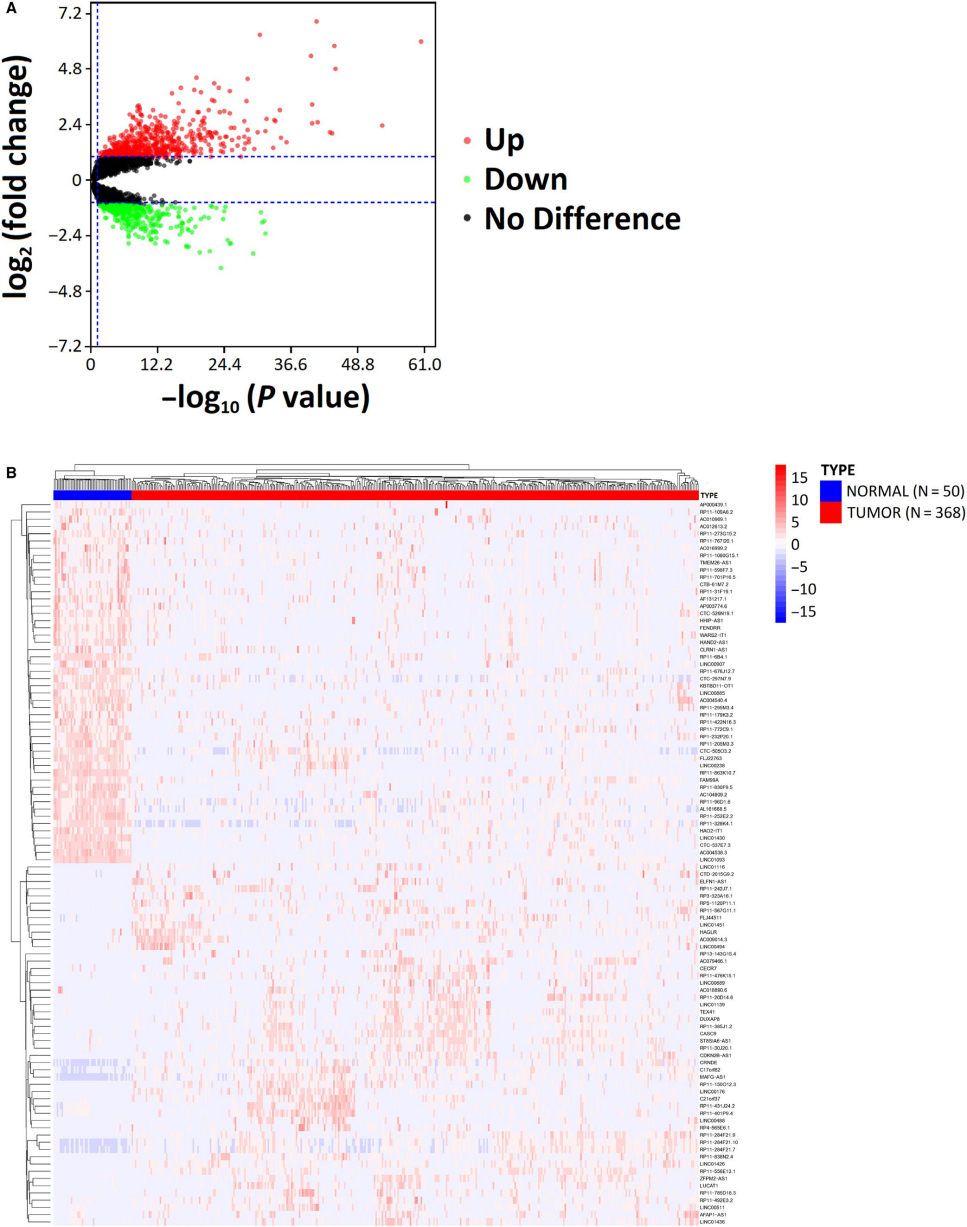
A, Volcano plot showing the differential expression of long noncoding RNAs (lncRNAs) in hepatocellular carcinoma (HCC); *X*‐axis represents log transformed *P* values adjusted by false discovery rate method; *Y*‐axis indicates the mean expression differences of lncRNAs between HCC and adjacent tissues. B, Heatmaps demonstrate differential expression of the top 50 upregulated lncRNAs and top 50 downregulated lncRNAs between HCC and adjacent tissues; *X*‐axis denotes differentially expressed lncRNAs and *Y*‐axis represents the sample types. The expression values are shown in line with color scale

Through the DElncRNAs analysis by R, large numbers of lncRNAs with abnormal expression in HCC were distinguished. With |log_2_(FC)| > 1.0 and *P* < .05, a total of 1048 DElncRNAs including 708 upregulated and 340 downregulated lncRNAs were obtained. Volcano plot was constructed to visually display the DElncRNAs that are found in HCC and adjacent tissues (Figure 1A). Among 1048 selected DElncRNAs, the top 50 upregulated and top 50 downregulated DElncRNAs (Table S1) were depicted as a heatmap (Figure 1B). The result of hierarchic cluster analysis showed distinguishable lncRNA expression patterns among tumor and normal tissue samples. The top 5 upregulated DElncRNAs were AFAP1‐AS1, AC079466.1, HAGLR, RP11‐556E13.1, and ST8SIA6‐AS1. While the top 5 downregulated DElncRNAs were LINC01093, AC016999.2, LINC00907, AP000439.1, and CTC‐297N7.9.

Receiver operating characteristic analysis was conducted on the top 50 up‐ and downregulated DElncRNAs to assess the diagnostic value of HCC. Following the threshold of AUC > 0.85, a total of 32 DElncRNAs (12 upregulated and 20 downregulated) with favorable diagnosis efficiency of HCC were selected (Table S2). These 32 DElncRNAs were then analyzed by univariate survival analysis to evaluate the prognostic value of HCC patients. With *P* < .05, four lncRNAs (RP11‐284F21.10, RP11‐284F21.7, MAFG‐AS1, and CTC‐297N7.9) were revealed to have significant association with OS, and three lncRNAs (RP11‐284F21.9, CDKN2B‐AS1, and CTC‐297N7.9) were shown obvious relevance to DFS of patients with HCC (Table S3). We noticed that the expression of CTC‐297N7.9 was significantly related to both OS and DFS of HCC patient. Therefore, we selected lncRNA CTC‐297N7.9, which have not been reported in HCC so far, for further analysis.

### 3.2 CTC‐297N7.9 was downregulated in HCC tissues and cell lines

**Figure 2:**
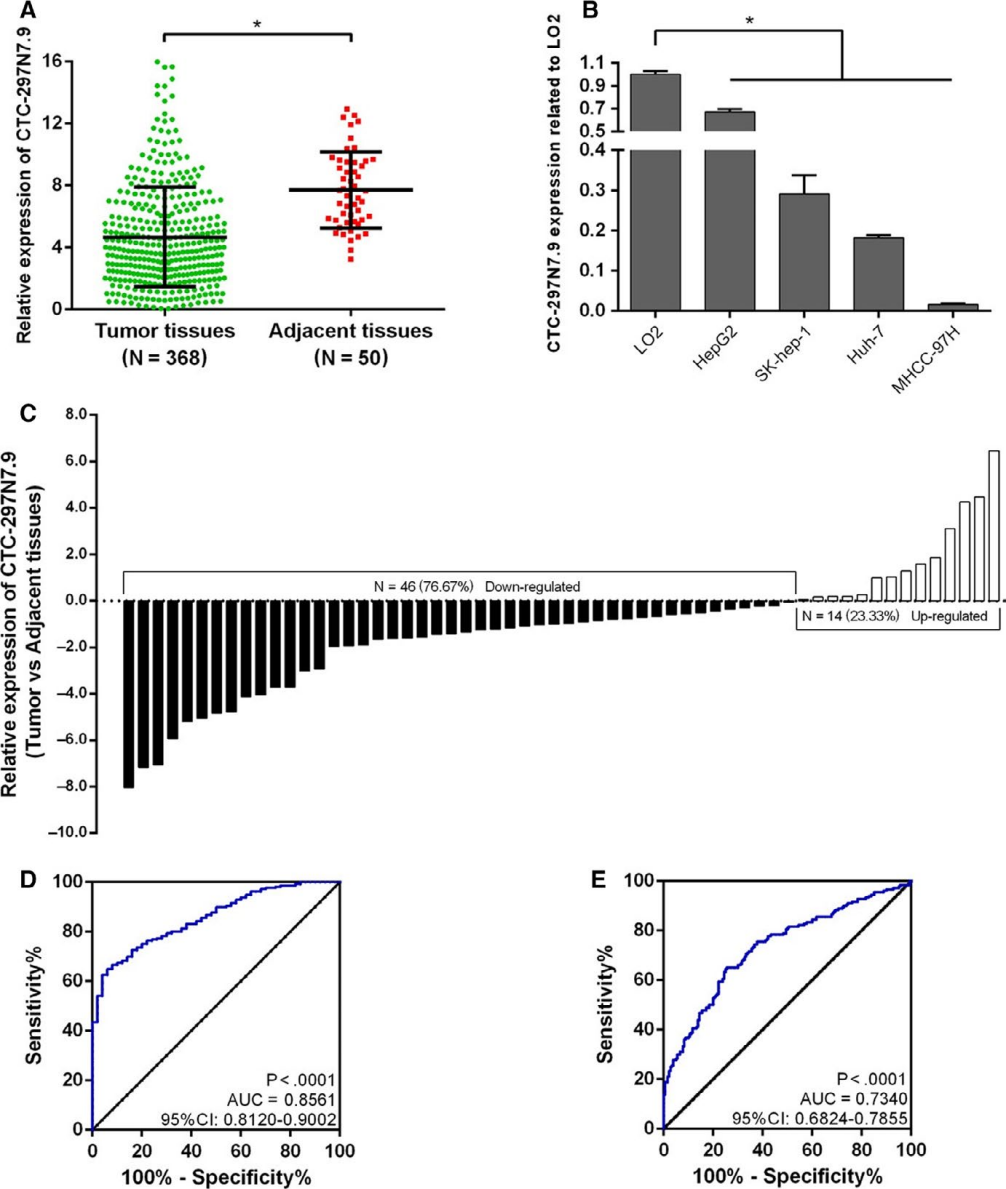
The expression of CTC‐297N7.9 in hepatocellular carcinoma (HCC) and the diagnostic value of CTC‐297N7.9 for HCC. A, CTC‐297N7.9 expression values in 368 HCC tissues and 50 adjacent tissues from the The Cancer Genome Atlas (TCGA) database. B, CTC‐297N7.9 expression values in four hepatoma cell lines and one normal liver cell line. (C) Comparison of CTC‐297N7.9 expression in 60 pairs of HCC and matched adjacent tissues obtained from 60 HCC patients in our center. Student *t* test, *P < .05. Receiver operating characteristic (ROC) curves were used to analyze the diagnostic performance of CTC‐297N7.9 to identify HCC and noncarcinoma tissues. The area under the ROC curve was shown with 95% confidence intervals. D, Significance of CTC‐297N7.9 in HCC with ROC analysis from the TCGA. E, Significance of CTC‐297N7.9 in HCC with ROC analysis from our center

The expression levels of CTC‐297N7.9 in 368 HCC and 50 adjacent tissues from the TCGA datasets were investigated using RNA‐sequencing data (FPKM normalization). Figure 2A showed that the expression of CTC‐297N7.9 in HCC tissues was significantly lower than that in adjacent tissues (*P* < .001). qRT‐PCR assay in four hepatoma cell lines and one normal liver cell line was performed to verify the downregulation of CTC‐297N7.9 in HCC. The results showed that the expression levels of CTC‐297N7.9 in HepG2, Sk‐hep‐1, Huh7, and MHCC‐97H were all significantly decreased, compared with that in LO2 (all *P* < .001) (Figure 2B). Moreover, the expression of CTC‐297N7.9 was further confirmed in 60 pairs of HCC and adjacent tissue specimens, which were surgically removed from 60 HCC patients in our center. As Figure 2C showed, the expression levels of CTC‐297N7.9 were markedly reduced in nearly 77% of HCC tissues (46/60) as compared to the corresponding adjacent tissues (*P* < .05).

### 3.3 Diagnostic value of CTC‐297N7.9 for HCC

In order to determine whether CTC‐297N7.9 can serve as a biomarker for HCC, ROC analysis was performed to evaluate the diagnostic significance of CTC‐297N7.9 for HCC. As shown in Figure 2D, with AUC at over 0.85 (*P* < .001), it was revealed that CTC‐297N7.9 has value in the diagnosis of HCC. Furthermore, the expression levels of CTC‐297N7.9 in HCC and corresponding adjacent tissues collected from our center were used to analyze its sensitivity and specificity for the diagnosis of HCC. The AUC of the ROC curve was nearly 0.74, which also revealed the diagnostic value of CTC‐297N7.9 in HCC (Figure 2E).

### 3.4 Relevance between CTC‐297N7.9 expression and clinicopathological characteristics

**Figure 3:**
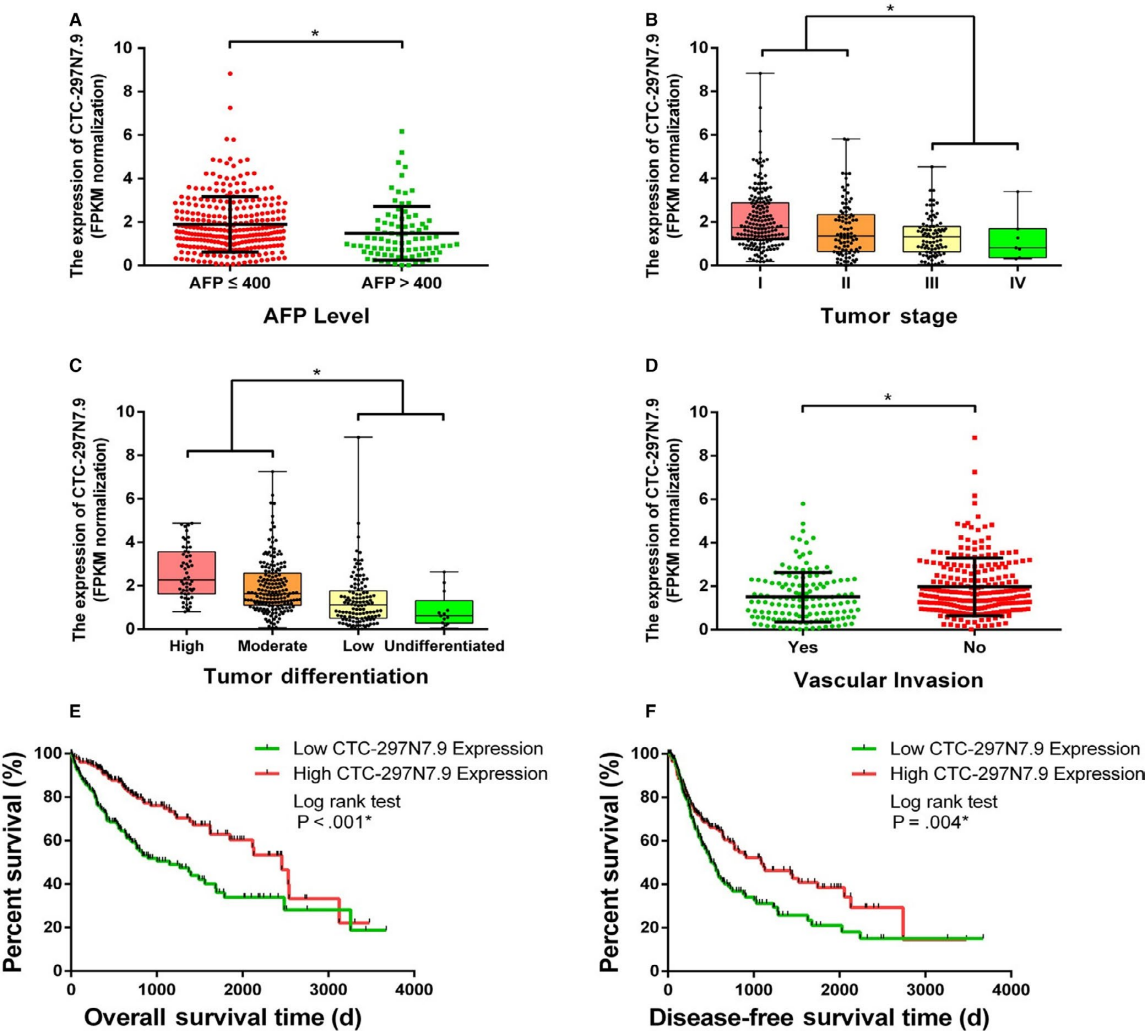
Clinical relevance and prognostic significance of CTC‐297N7.9 in hepatocellular carcinoma (HCC). A, CTC‐297N7.9 expression levels in HCC patients from the The Cancer Genome Atlas (TCGA) database with low and high levels of serum alpha‐fetal protein (AFP). B, CTC‐297N7.9 expression levels in HCC patients from the TCGA database at different tumor stages. C, CTC‐297N7.9 expression levels in HCC patients from the TCGA database with different degrees of tumor differentiation. D, CTC‐297N7.9 expression levels in HCC patients from the TCGA database with and without vascular invasion. E, Kaplan‐Meier (K‐M) curves for OS of 368 HCC patients from the TCGA with high and low CTC‐297N7.9 expression. F, K‐M curves for disease‐free survival of 368 HCC patients from the TCGA with high and low CTC‐297N7.9 expression. Student *t* test and log rank test, *P < .05

The clinical information of 368 HCC patients from the TCGA database was analyzed. The expression level of CTC‐297N7.9 in HCC tissues was labeled as low or high in relation to the median value. Correlation analysis revealed that CTC‐297N7.9 expression was significantly related to serum AFP level (*P* = .007), tumor node metastasis (TNM) stage (*P* = .011), tumor differentiation (*P* < .001), and vascular invasion (*P* = .024) (Table 1). Patients with serum AFP higher than 400 μg/L had lower CTC‐297N7.9 expression levels in HCC tissues, compared to those with AFP lower than 400 μg/L (Figure 3A). On the contrary, low expression level of CTC‐297N7.9 in HCC was found to be positively correlated with advanced tumor stage (Figure 3B) and poor tumor differentiation (Figure 3C). Moreover, the expression of CTC‐297N7.9 in patients without vascular invasion was significantly higher than that of the patients with vascular invasion (Figure 3D). However, CTC‐297N7.9 expression level had no significant association with other characteristics, such as age, gender, race, viral hepatitis infection, Child‐Pugh classification as well as liver cirrhosis (*P* > .05).

**Table 1:** Relationship between CTC‐297N7.9 expression and clinicopathological characteristics in 368 HCC patients from the TCGA database

Clinicopathological characteristics	CTC‐297N7.9 expression^a^		*P* value^b^
	Low (N = 184)	High (N = 184)	
Age (y)			
≤60	88	89	.917
>60	96	95	
Gender			
Male	122	128	.503
Female	62	56	
Race			
Asian	85	78	.399
White	88	99	
Black and African American	11	7	
Child‐Pugh			
A	163	171	.334
B	20	12	
C	1	1	
Hepatitis virus infection			
HBV	46	51	.653
HCV	32	24	
Others	8	10	
No infection	98	99	
Alcoholic liver			
Yes	61	56	.576
No	123	128	
Cirrhosis			
Yes	70	66	.666
No	114	118	
AFP (μg/L)			
≤400	130	152	.007*
>400	54	32	
Tumor stage			
I	76	107	.011*
II	51	40	
III	52	35	
IV	5	2	
Tumor differentiation			
High	12	42	<.001*
Moderate	79	100	
Low	83	39	
Undifferentiated	10	3	
Vascular invasion			
Yes	81	60	.024*
No	103	124	

Abbreviations: HBV, hepatitis B virus; HCV, hepatitis C virus; AFP, alpha‐fetal protein; HCC, hepatocellular carcinoma.

a The median expression level of CTC‐297N7.9 was used as the cutoff. Low CTC‐297N7.9 expression among the 184 patients was defined as a value below the 50th percentile; while high CTC‐297N7.9 expression among the 184 patients was defined as a value above the 50th percentile.

b Chi‐square test.

* *P* < .05.

### 3.5 Correlation with CTC‐297N7.9 expression and survival of HCC patients

Kaplan‐Meier curves for OS and DFS were plotted to analyze the prognosis of HCC patients with high and low CTC‐297N7.9 expression. Figure 3E,F separately depicted the OS and DFS curve of 368 HCC patients from the TCGA database. The median OS of HCC patients with low CTC‐297N7.9 expression was significantly shorter than that of HCC patients with high CTC‐297N7.9 expression. Same pattern was observed in the DFS of HCC patients as well. Log rank test showed that there is statistic difference between the two groups in both OS (*P* < .001) and DFS (*P* = .004). Univariate regression analysis revealed that TNM stage, tumor differentiation, vascular invasion, and CTC‐297N7.9 expression were prognostic factors for both OS and DFS (Table S4); multivariate regression analysis showed that the expression of CTC‐297N7.9 is an independent prognostic factor in HCC (Table S5).

**Figure 4:**
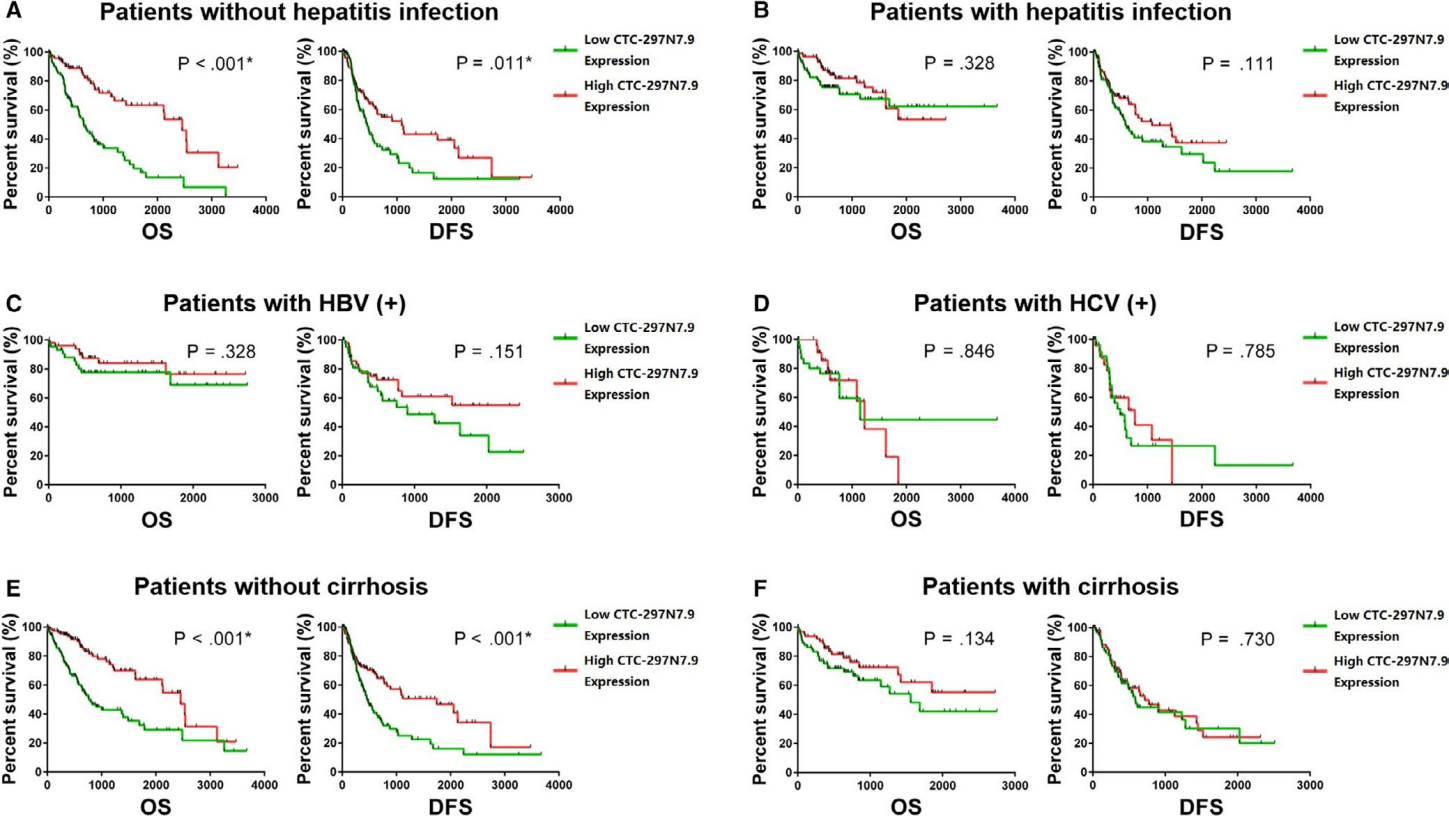
Relationship between the expression levels of CTC‐297N7.9 and survival of hepatocellular carcinoma (HCC) patients from the The Cancer Genome Atlas (TCGA) database. A, Overall survival (OS) and disease‐free survival (DFS) curves of HCC patients without hepatitis virus infection. B, OS and DFS curves of HCC patients with hepatitis virus infection. C, OS and DFS curves of HCC patients with HBV positive. D, OS and DFS curves of HCC patients with HCV positive. E, OS and DFS curves of HCC patients without cirrhosis. F, OS and DFS curves of HCC patients with cirrhosis. Log rank test, *P < .05

Subgroup analysis was then carried out to further explore the correlation between CTC‐297N7.9 expression and prognosis of HCC patients with different characteristics. Three hundred and sixty‐eight HCC patients from the TCGA were divided into several subgroups according to their clinical features such as race, gender, age, hepatitis virus infection, alcoholic liver, cirrhosis, and serum AFP level. The results of survival analysis for subgroups of race, gender, age, level of alcohol consumption, and serum AFP are consistent with the previous conclusions (Figure S1). Besides, statistical difference was observed in both OS and DFS of patients without hepatitis virus infection when divided into two groups of high or low CTC‐297N7.9 expression (Figure 4A). However, we found no significant difference in OS and DFS of the subgroup of patients with viral hepatitis (Figure 4B), including hepatitis B virus (HBV) (Figure 4C) and hepatitis C virus (HCV) (Figure 4D). Similarly, for patients without cirrhosis, those with low CTC‐297N7.9 expression have shorter OS and DFS than that of patients with high CTC‐297N7.9 expression (Figure 4E). Lastly, for the subgroup of patients with cirrhosis, although the median OS and DFS of patients with high expression of CTC‐297N7.9 were slightly longer, neither OS nor DFS had significant statistic difference (Figure 4F).

### 3.6 Clinical relevance and prognostic significance of CTC‐297N7.9 in HCC patients from our center

**Figure 5:**
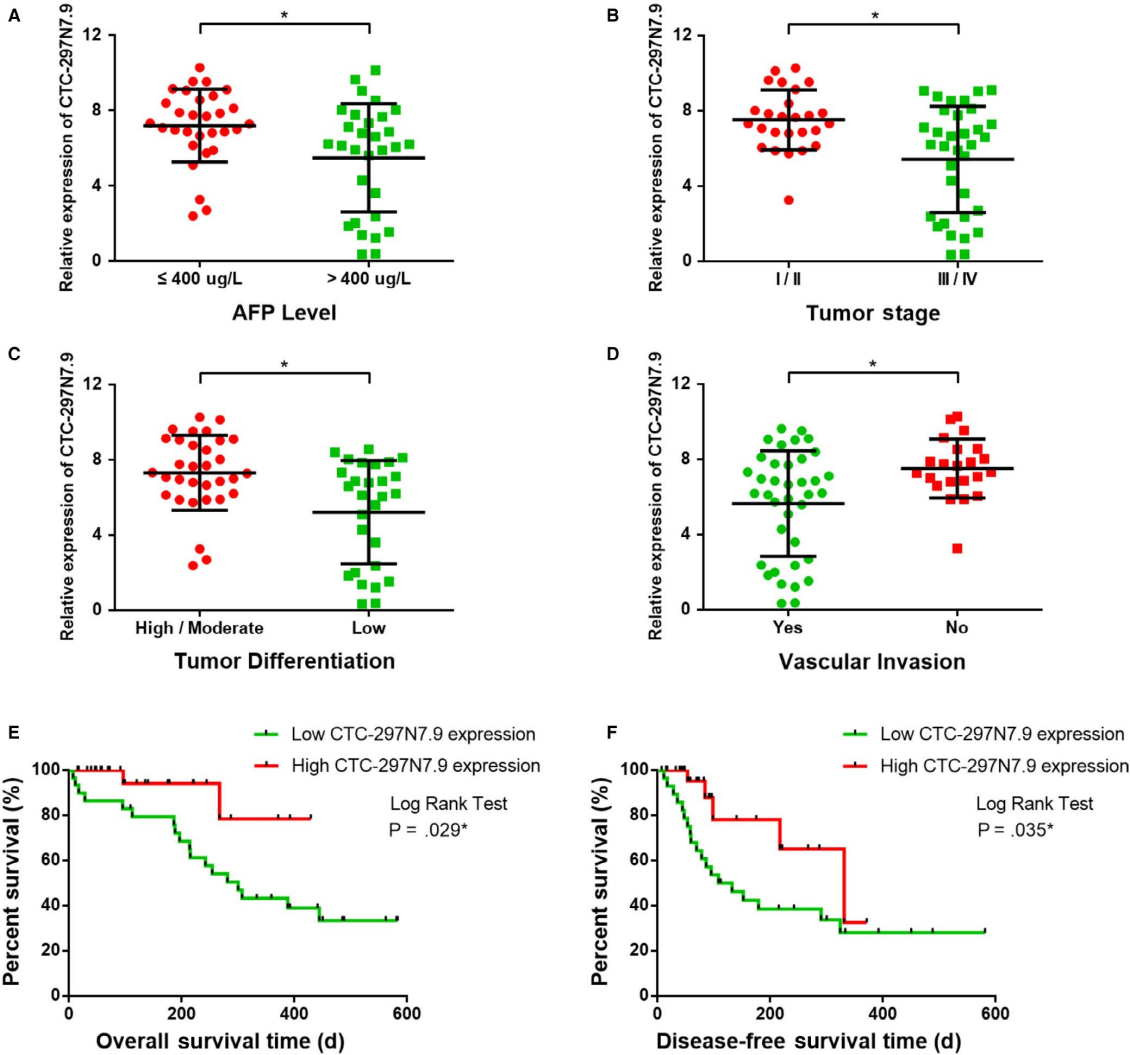
Clinical relevance and prognostic significance of CTC‐297N7.9 in 60 hepatocellular carcinoma (HCC) patients from our center. A, CTC‐297N7.9 expression of HCC patients with high and low level of serum alpha‐fetal protein (AFP). B, CTC‐297N7.9 expression of HCC patients with different degrees of tumor differentiation. C, CTC‐297N7.9 expression of HCC patients at early and advanced tumor stage. D, CTC‐297N7.9 expression of HCC patients with and without vascular invasion. E, Survival curves for overall survival of HCC patients from our center. F, Survival curves for disease‐free survival of HCC patients from our center. Student *t* test and log rank test, *P < .05

The clinical information and survival prognosis of 60 HCC patients from our center, whose tissue specimens were included in this study, were further explored to verify the conclusion drawn from the bioinformatics analysis. By analyzing the clinicopathological characteristics, we found that in addition to AFP level (*P* = .039), tumor stage (*P* = .037), and differentiation (*P* = .038), vascular invasion (*P* = .032) was also significantly associated with CTC‐297N7.9 expression (Table 2). Consistent with the results of bioinformatics analysis, patients with high AFP level (Figure 5A), advanced tumor stage (Figure 5B), and poor tumor differentiation (Figure 5C) had lower CTC‐297N7.9 expression level in their tumor tissues. Furthermore, the expression of CTC‐297N7.9 in tumors from patients with tumor vascular invasion (Figure 5D) was also downregulated, compared to that of patients without tumor vascular invasion.

**Table 2:** Relationship between CTC‐297N7.9 expression and clinicopathological characteristics in 60 HCC patients from our center

Clinicopathological characteristics	CTC‐297N7.9 expression^a^		*P* value^b^
	Low (N = 30)	High (N = 30)	
Age (y)			
≤60	17	23	.100
>60	13	7	
Gender			
Male	27	27	1.000
Female	3	3	
Child‐Pugh			
A	25	27	.448
B/C	5	3	
HBs antigen			
Positive	21	27	.053
Negative	9	3	
Cirrhosis			
Yes	13	16	.438
No	17	14	
AFP (μg/L)			
≤400	11	19	.039*
>400	19	11	
Tumor stage			
I/II	9	17	.037*
III/IV	21	13	
Tumor size (cm)			
≤5	8	10	.573
>5	22	20	
Tumor differentiation			
High/moderate	12	20	.038*
Low/undifferentiated	18	10	
Vascular invasion			
Yes	23	15	.032*
No	7	15	

Abbreviations: HBs antigen, hepatitis B surface antigen; AFP, alpha‐fetal protein; HCC, hepatocellular carcinoma.

a The median expression level of CTC‐297N7.9 was used as the cutoff. Low CTC‐297N7.9 expression among the 30 patients was defined as a value below the 50th percentile; while high CTC‐297N7.9 expression among the 30 patients was defined as a value above the 50th percentile.

b Chi‐square test.

* *P* < .05.

The HCC patients from our center had all undergone clinical follow‐up for more than 1 year. Survival curves showed that median OS and median DFS of patients with low CTC‐297N7.9 expression were all obviously shorter than those with high CTC‐297N7.9 expression (Figure 5E,F), which were consistent with the results of bioinformatics analysis in the TCGA database. 6‐month and 1‐year OS and DFS ROC curves were also carried out to assess the efficiency of CTC‐297N7.9 in prognostic prediction (Figure S2). The AUCs of 6‐month and 1‐year OS were 0.735 and 0.773, respectively, whereas the AUCs of 6‐month and 1‐year DFS were 0.766 and 0.810. All the AUCs were over 0.70, which showed that CTC‐297N7.9 has prognostic value in OS and DFS of HCC patients.

### 3.7 Establishment of survival prognostic models for HCC

**Figure 6:**
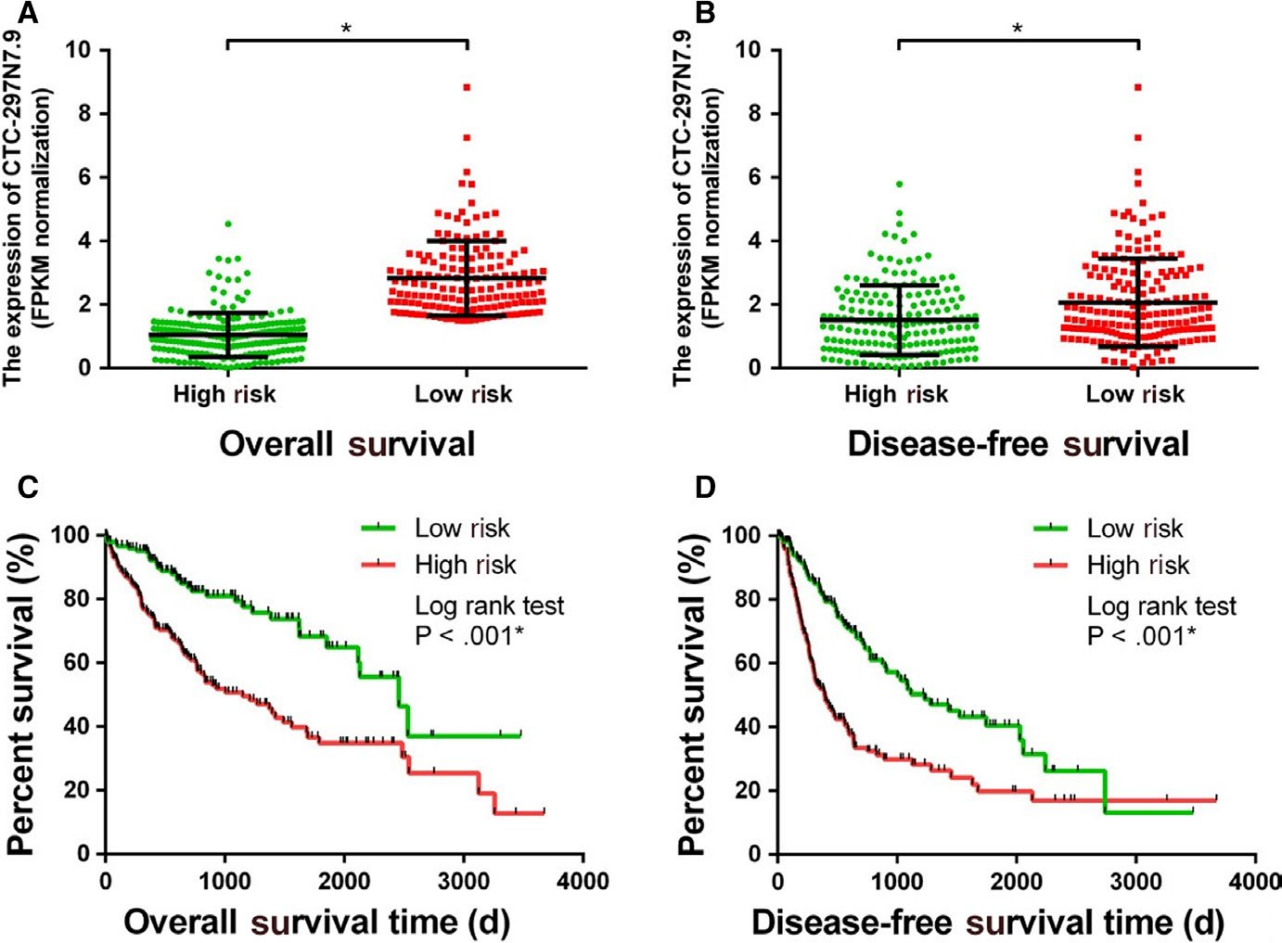
Survival prognostic models for hepatocellular carcinoma (HCC). A, The expression level of CTC‐297N7.9 in patients of high‐ and low‐risk groups by the overall survival (OS) model. B, The expression level of CTC‐297N7.9 in patients of high‐ and low‐risk groups by the disease‐free survival (DFS) model. C, Kaplan‐Meier (K‐M) curves for OS of patients in high‐ and low‐risk group. D, K‐M curves for DFS of patients in high‐ and low‐risk group

We attempted to build the survival prognostic models for HCC by combining the expression level of CTC‐297N7.9 with other clinicopathological characteristics of HCC patients from the TCGA to further establish the prognostic significance of CTC‐297N7.9 in HCC. We defined the clinicopathological characteristics of male, age over 60, Child‐Pugh class in B or C, with hepatitis virus infection, alcohol liver, cirrhosis, serum AFP > 400 μg/L, advanced TNM stage, low tumor differentiation, vascular invasion, and high CTC‐297N7.9 expression as 1 in the score of clinical features; and a score of 0 was designated to the characteristics of female, age ≤60, Child‐Pugh class A, no viral hepatitis, no alcohol liver, no cirrhosis, AFP ≤ 400 μg/L, early TNM stage, and low CTC‐297N7.9 expression. According to the multivariate regression analysis, survival prognostic models for both OS and DFS were established. The formula of the OS prognostic model is given as: Risk score = −0.5281 × *S*_hepatitis_ + 0.6477 × *S*_TNM_ − 0.7820 × *S*_CTC‐297N7.9_; while the formula for the DFS prognostic model is given as: risk score = 0.6293 × *S*_TNM_ + 0.3445 × *S*_vascular_ − 0.3330 × *S*_CTC‐297N7.9_. The risk scores of OS and DFS were separately calculated, and 368 HCC patients from the TCGA were divided into high‐ and low‐risk groups. Figure 6A showed that the expression of CTC‐297N7.9 in high‐risk group was significantly lower than that in low‐risk group (*P* < .001), according to the classification of the OS prognostic model. Meanwhile, Figure 6B depicted a similar result through the DFS prognostic model that low‐risk group had higher CTC‐297N7.9 expression (*P* < .001). The survival time of both groups was also compared. Using the K‐M analysis, the median OS in low‐risk group was shown to be significantly longer than that of the high‐risk group (Figure 6C). The DFS was obviously shortened in high‐risk group as compared to the low‐risk group (Figure 6D). These results indicated good performance in prognostic prediction of both the OS and DFS models.

## 4 DISCUSSION

Hepatocellular carcinoma is a common malignant tumor, which ranks the fifth most frequent cancer and the fourth leading reason of cancer‐associated mortality worldwide.^19^ Due to the highly aggressive nature of the tumor, there are still over 80% of recurrence rate among patients within 5 years after curative surgical resection of primary HCC.^20^ Although a number of tumor markers such as AFP have been exploited for HCC diagnosis, the high heterogeneity and complex mechanism of the tumor have made those biomarkers insensitive and not specific enough to evaluate the prognosis of HCC patients.^21^ Moreover, the predictive effect of conventional clinical tumor staging systems such as TNM and Barcelona Clinic Liver Cancer (BCLC) may vary for patients with different pathogeny and tumor morphology, thus limiting their contribution to the prediction of prognosis for HCC patients.^22^ So, it is vital to discover a new biomarker which could effectively predict the prognosis of HCC patients. In our study, we investigated the expression level of CTC‐297N7.9 in HCC cell lines and tissues and assessed the prognostic significance of this lncRNA for HCC patients' survival. To the best of our knowledge, it is the first time the dysregulated expression pattern of CTC‐297N7.9 is reported in HCC. More importantly, this novel lncRNA was verified to be useful in diagnosing HCC and predicting the survival of HCC patients, showing its potential as a novel biomarker for HCC.

The novel type of RNA molecule called the lncRNA has become one of the hot topics in life science research. This class of noncoding RNAs has been identified to participate in the regulation of gene expression at transcription, posttranscription, and epigenetics levels, which influence important biological activities such as genomic imprinting, X‐chromosome inactivation, chromatin modification, and transcriptional activation and inhibition.^23^, ^24^, ^25^ Long noncoding RNAs are generally classified as five broad categories based on their genomic proximity between neighboring transcripts: sense, antisense, bidirectional, intronic, and intergenic.^26^ CTC‐297N7.9, also named as AC015908.3, ENSG00000264016, or OTTHUMG00000178048.2, is a type of long intergenic noncoding RNA with a length of 457nt and is located in chromosome 17p13.1.

Many lncRNAs were previously proven to be involved in the development and progression of HCC, including Highly Upregulated in Liver Cancer (HULC), H19, maternally expressed gene 3 (MEG3), lncRNA‐LET, HOTAIR, and MALAT1.^27^ For instance, HULC was confirmed to inhibit the activity of a series of microRNAs including microRNA‐372. Its expression level is positively correlated with those of hepatitis B virus X protein in clinical HCC tissues, similar to oncogene.^28^, ^29^ HOX Antisense Intergenic RNA and MALAT1 play important roles in HCC progression and serve as the biomarkers for predicting tumor recurrence following liver transplantation.^30^, ^31^ Other than that, MEG3, the first lncRNA that was suggested to function as a tumor suppressor gene, had been corroborated to interact with P53 and inhibit the proliferation of many hepatoma cell lines in vitro.^32^, ^33^, ^34^ It was reported that the downregulated expression of MEG3 in HCC was modulated by microRNA‐29 via the methylation machinery.^35^ H19 could suppress not only HCC progression by decreasing the expression of markers for epithelial‐to‐mesenchymal transition, but also activate miR‐200 family by increasing histone acetylation.^36^ Recently, lnc‐FTX was reported to inhibit the proliferation and metastasis of HCC by binding to MCM2 and miR‐374a.^37^ Moreover, the downregulation of lncRNA GAS5‐AS1 correlates to poor survival of HCC patients, making it a potential prognostic and diagnostic marker in HCC.^38^ Nowadays, the TCGA has provided a convenient platform for researchers to discover and identify novel lncRNAs and explore their potential for survival prediction. Current studies have reported that a number of novel lncRNAs such as LINC01559,^39^ RP11‐486O12.2,^40^ and SNHG4,^41^ which have not been reported in HCC, were identified through differential gene expression analysis and were all proven to be prognostic biomarkers for HCC.

Recent research pointed out that lncRNA CTC‐297N7.9 was differentially expressed in HCC and normal liver tissues. According to the TCGA database, its expression is significantly associated with the survival of HCC patients.^39^, ^40^, ^41^ CTC‐297N7.9 was also reported to participate in the construction of prognostic models that identified the role of lncRNA as a protective factor in HCC.^42^, ^43^, ^44^ These results, however, were not verified by molecular experiments yet. In our study, we identified the aberrant expression of CTC‐297N7.9 using not only gene expression matrix of the TCGA but also by HCC cell lines and tissue samples from our center. The diagnostic and prognostic value of CTC‐297N7.9 were evaluated by analyzing both TCGA cohort and our cohort. We detected the decreased expression and the diagnostic significance of CTC‐297N7.9 in HCC. We also found that low expression level of CTC‐297N7.9 is closely associated with the clinicopathological characteristics indicating poor prognosis of HCC patients, such as high serum AFP level, advanced tumor stage, poor differentiation, and vascular invasion. Furthermore, CTC‐297N7.9 expression level was identified to be positively correlated to the patients' survival. Cox analysis suggested that CTC‐297N7.9 expression is an independent prognostic factor in HCC for both OS and DFS. Subgroup analysis revealed that the survival differences between patients with high and low CTC‐297N7.9 expression level in tumor tissues cannot be distinguished. However, in patients without viral hepatitis infection or without cirrhosis, CTC‐297N7.9 had better predictive effect for the patients' outcome with a higher level of specificity. Moreover, significant values were identified in the OS and DFS prognostic models that were constructed using the expression level of CTC‐297N7.9 and other clinical characteristics. CTC‐297N7.9 has been proven to be negatively correlated to risk score, indicating its role as a protective factor in HCC. All these results suggested the possible function of CTC‐297N7.9 as a tumor suppressor gene. Long noncoding RNA CTC‐297N7.9 can serve as a potential prognosis predictive biomarker for HCC patients.

## 5 CONCLUSION

Our study demonstrated the clinical significance of a novel lncRNA CTC‐297N7.9, which expression was downregulated in HCC cell lines and tissues, in diagnosing HCC and predicting the prognosis of HCC patients. Low expression of CTC‐297N7.9 was significantly associated with poor prognosis, especially among patients without viral hepatitis or cirrhosis. All these indicated that CTC‐297N7.9 can be a potential prognostic marker for HCC. However, the underlying molecular mechanism of CTC‐297N7.9 in HCC is still unclear. Further research is needed to clarify the role of CTC‐297N7.9 in the pathogenesis and development of HCC.

## CONFLICT OF INTERESTS

None declared.

## ETHICS APPROVAL AND CONSENT TO PARTICIPATE

This study was approved by the Ethics Committee of Sun Yat‐sen Memorial Hospital, Sun Yat‐sen University. Informed consent was signed by all the patients whose tissue specimens were used in our study.
