# Supplementary figures and images for: Low expression of long noncoding RNA CTC‐297N7.9 predicts poor prognosis in patients with hepatocellular carcinoma
Source: Cancer Med. 2019 Nov 1;8(18):7679–92. doi: 10.1002/cam4.2618 (PMC6912069; doi:10.1002/cam4.2618)

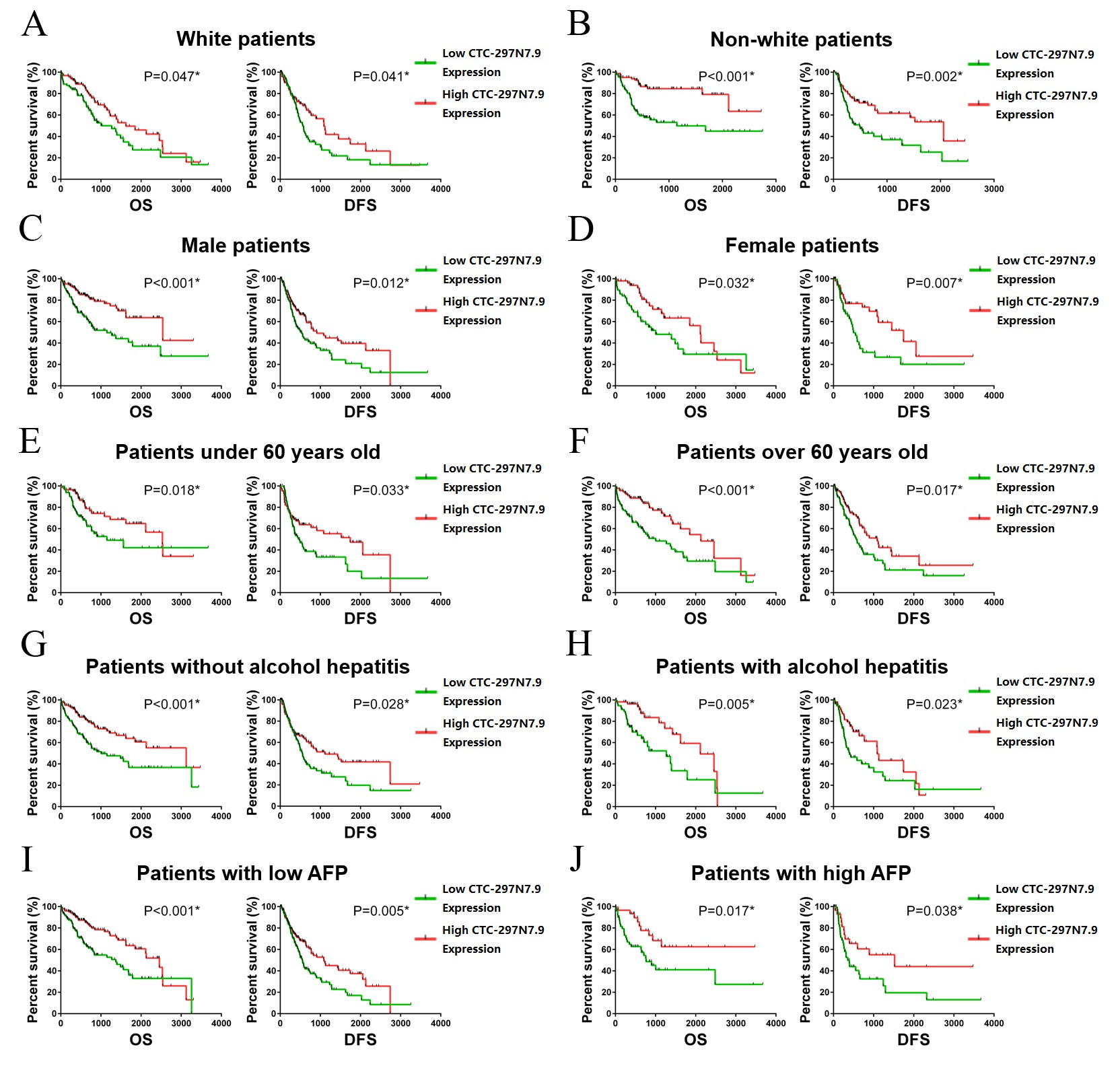

Supplement: Supplementary file 1 [file CAM4-8-7679-s001.tif]

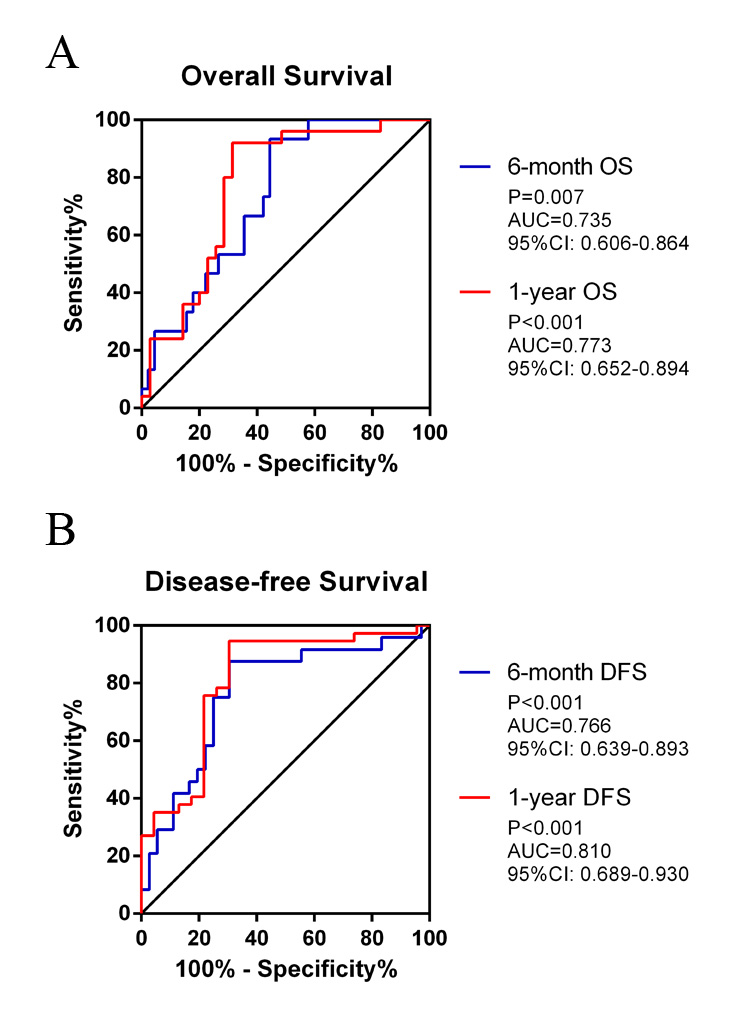

Supplement: Supplementary file 2 [file CAM4-8-7679-s002.tif]
