# Supplementary material for: Low expression of long noncoding RNA CTC‐297N7.9 predicts poor prognosis in patients with hepatocellular carcinoma
Source: Cancer Med. 2019 Nov 1;8(18):7679–92. doi: 10.1002/cam4.2618 (PMC6912069; doi:10.1002/cam4.2618)
Supplement: Supplementary file 3 [file CAM4-8-7679-s003.docx]

**Figure S1.** Relationship between the expression levels of CTC-297N7.9 and survival of HCC patients from the TCGA database. (A) OS and DFS curves of white HCC patients. (B) OS and DFS curves of non-white HCC patients. (C) OS and DFS curves of male HCC patients. (D) OS and DFS curves of female HCC patients. (E) OS and DFS curves of HCC patients under 60 years old. (F) OS and DFS curves of HCC patients over 60 years old. (G) OS and DFS curves of HCC patients without alcoholic hepatitis. (H) OS and DFS curves of HCC patients with alcoholic hepatitis. (I) OS and DFS curves of HCC patients with serum AFP lower than 400μg/L. (J) OS and DFS curves of HCC patients with serum AFP higher than 400μg/L. Log rank test, *P<0.05

**Figure S2.** ROC analysis for prognosis of the 60 HCC patients in our center. (A) 6-month and 1-year OS ROC curves. (B) 6-month and 1-year DFS ROC curves
