# Supplementary material for: Low expression of long noncoding RNA CTC‐297N7.9 predicts poor prognosis in patients with hepatocellular carcinoma
Source: Cancer Med. 2019 Nov 1;8(18):7679–92. doi: 10.1002/cam4.2618 (PMC6912069; doi:10.1002/cam4.2618)
Supplement: Supplementary file 4 [file CAM4-8-7679-s004.docx]

**Supplementary Materials**

**Table S1.** Top 50 up-regulated and down-regulated differentially expressed lncRNAs in HCC

| **lncRNA** | **Log_2_(fold change)** | **P value** | **Regulation** |
| --- | --- | --- | --- |
| AFAP1-AS1 | 6.9199 | 1.2628E-41 | Up-regulated |
| AC079466.1 | 6.3745 | 3.1589E-31 | Up-regulated |
| HAGLR | 6.0950 | 7.8345E-61 | Up-regulated |
| RP11-556E13.1 | 5.8741 | 6.9735E-45 | Up-regulated |
| ST8SIA6-AS1 | 5.4710 | 1.1476E-40 | Up-regulated |
| LINC00176 | 4.8508 | 3.8185E-45 | Up-regulated |
| AC018890.6 | 4.5055 | 9.5362E-20 | Up-regulated |
| RP11-385J1.2 | 4.4305 | 4.9388E-29 | Up-regulated |
| RP11-242J7.1 | 4.2348 | 5.5095E-23 | Up-regulated |
| AC009014.3 | 4.0800 | 8.0010E-17 | Up-regulated |
| RP13-143G15.4 | 4.0449 | 9.6229E-24 | Up-regulated |
| RP11-431J24.2 | 3.9777 | 2.7362E-19 | Up-regulated |
| ZFPM2-AS1 | 3.9277 | 7.6172E-26 | Up-regulated |
| LUCAT1 | 3.8616 | 1.4399E-20 | Up-regulated |
| CECR7 | 3.8167 | 2.4099E-15 | Up-regulated |
| RP5-1120P11.1 | 3.5336 | 1.1951E-22 | Up-regulated |
| RP11-284F21.9 | 3.4797 | 4.4710E-23 | Up-regulated |
| RP11-284F21.10 | 3.4742 | 8.1572E-29 | Up-regulated |
| RP11-492E3.2 | 3.4019 | 1.3871E-16 | Up-regulated |
| CDKN2B-AS1 | 3.3510 | 7.0623E-41 | Up-regulated |
| LINC00488 | 3.2777 | 2.9794E-09 | Up-regulated |
| LINC01139 | 3.2368 | 4.7159E-09 | Up-regulated |
| LINC00689 | 3.2036 | 2.7237E-09 | Up-regulated |
| RP11-30J20.1 | 3.0983 | 2.2193E-09 | Up-regulated |
| LINC00511 | 3.0938 | 6.1563E-22 | Up-regulated |
| LINC01436 | 3.0877 | 9.1648E-12 | Up-regulated |
| CRNDE | 3.0867 | 4.5673E-35 | Up-regulated |
| RP11-284F21.7 | 3.0605 | 8.9566E-25 | Up-regulated |
| CASC9 | 3.0510 | 6.3144E-09 | Up-regulated |
| RP4-565E6.1 | 3.0469 | 3.1403E-17 | Up-regulated |
| LINC01451 | 3.0175 | 4.8723E-24 | Up-regulated |
| TEX41 | 3.0024 | 4.4828E-20 | Up-regulated |
| LINC01116 | 2.9801 | 5.4612E-20 | Up-regulated |
| DUXAP8 | 2.9396 | 6.9863E-26 | Up-regulated |
| RP11-20D14.6 | 2.9083 | 1.3042E-09 | Up-regulated |
| ELFN1-AS1 | 2.9021 | 1.3661E-09 | Up-regulated |
| RP11-785D18.3 | 2.8871 | 1.5899E-07 | Up-regulated |
| RP3-323A16.1 | 2.8796 | 3.0875E-13 | Up-regulated |
| RP11-476K15.1 | 2.8781 | 9.9617E-10 | Up-regulated |
| RP11-401P9.4 | 2.8292 | 5.1866E-14 | Up-regulated |
| RP11-150O12.3 | 2.8087 | 2.7145E-11 | Up-regulated |
| LINC00494 | 2.8018 | 3.9327E-08 | Up-regulated |
| FLJ44511 | 2.7669 | 3.6802E-21 | Up-regulated |
| C21orf37 | 2.7633 | 3.0264E-12 | Up-regulated |
| RP11-838N2.4 | 2.7607 | 1.7982E-16 | Up-regulated |
| RP11-567G11.1 | 2.7544 | 3.3603E-08 | Up-regulated |
| C17orf82 | 2.7330 | 1.3101E-32 | Up-regulated |
| CTD-2015G9.2 | 2.6745 | 1.0586E-08 | Up-regulated |
| LINC01426 | 2.6634 | 4.0054E-19 | Up-regulated |
| MAFG-AS1 | 2.6585 | 6.3505E-34 | Up-regulated |
| CTC-505O3.2 | -1.9560 | 1.1883E-09 | Down-regulated |
| RP11-767I20.1 | -1.9589 | 1.5225E-08 | Down-regulated |
| RP11-273G15.2 | -1.9965 | 3.2160E-10 | Down-regulated |
| AC010969.1 | -1.9980 | 3.6142E-06 | Down-regulated |
| FLJ22763 | -2.0104 | 4.7246E-08 | Down-regulated |
| RP11-96D1.6 | -2.0119 | 4.7298E-15 | Down-regulated |
| RP11-1080G15.1 | -2.0155 | 4.2549E-17 | Down-regulated |
| AC012613.2 | -2.0164 | 4.9992E-10 | Down-regulated |
| RP11-179K3.2 | -2.0265 | 1.3241E-09 | Down-regulated |
| LINC00885 | -2.0426 | 5.8407E-07 | Down-regulated |
| AP003774.6 | -2.0521 | 3.6096E-18 | Down-regulated |
| RP11-772C9.1 | -2.0534 | 1.1218E-10 | Down-regulated |
| FAM99A | -2.0646 | 9.0631E-08 | Down-regulated |
| RP11-701P16.5 | -2.0772 | 3.0884E-18 | Down-regulated |
| WARS2-IT1 | -2.0830 | 8.9205E-12 | Down-regulated |
| RP11-31F19.1 | -2.1192 | 3.6655E-12 | Down-regulated |
| HHIP-AS1 | -2.1467 | 2.4281E-10 | Down-regulated |
| AL161668.5 | -2.1514 | 5.3542E-17 | Down-regulated |
| CLRN1-AS1 | -2.1728 | 2.3237E-07 | Down-regulated |
| RP11-109A6.2 | -2.1766 | 1.7684E-12 | Down-regulated |
| RP11-328K4.1 | -2.1792 | 2.5864E-11 | Down-regulated |
| TMEM26-AS1 | -2.1818 | 6.7104E-19 | Down-regulated |
| CTB-61M7.2 | -2.1877 | 9.8816E-12 | Down-regulated |
| CTC-526N19.1 | -2.2141 | 2.1145E-32 | Down-regulated |
| LINC01430 | -2.2632 | 9.1646E-17 | Down-regulated |
| LINC00238 | -2.2647 | 1.2130E-08 | Down-regulated |
| KBTBD11-OT1 | -2.3312 | 5.2747E-11 | Down-regulated |
| RP1-232P20.1 | -2.3506 | 2.4703E-07 | Down-regulated |
| RP11-205M3.3 | -2.3640 | 3.1989E-09 | Down-regulated |
| RP11-6B4.1 | -2.3642 | 2.2674E-07 | Down-regulated |
| HAND2-AS1 | -2.4042 | 5.2902E-10 | Down-regulated |
| RP11-422N16.3 | -2.4066 | 2.4743E-12 | Down-regulated |
| HAO2-IT1 | -2.4496 | 2.3853E-11 | Down-regulated |
| AC004540.4 | -2.4924 | 1.0825E-11 | Down-regulated |
| RP11-863K10.7 | -2.5110 | 2.0801E-25 | Down-regulated |
| RP11-830F9.5 | -2.5337 | 2.1583E-11 | Down-regulated |
| AC004538.3 | -2.5561 | 5.0005E-14 | Down-regulated |
| RP11-252E2.2 | -2.6363 | 1.8921E-14 | Down-regulated |
| RP11-295M3.4 | -2.6532 | 7.9350E-26 | Down-regulated |
| AF131217.1 | -2.6597 | 4.6013E-26 | Down-regulated |
| AC104809.2 | -2.6647 | 2.0784E-07 | Down-regulated |
| RP11-676J12.7 | -2.6648 | 4.0383E-12 | Down-regulated |
| RP11-598F7.3 | -2.7592 | 6.0132E-18 | Down-regulated |
| CTC-537E7.3 | -2.7948 | 5.8819E-18 | Down-regulated |
| FENDRR | -2.8252 | 3.1599E-18 | Down-regulated |
| CTC-297N7.9 | -2.9019 | 1.015E-18 | Down-regulated |
| AP000439.1 | -3.0182 | 3.7053E-22 | Down-regulated |
| LINC00907 | -3.0539 | 1.9198E-20 | Down-regulated |
| AC016999.2 | -3.1094 | 4.6078E-30 | Down-regulated |
| LINC01093 | -3.7005 | 2.8064E-24 | Down-regulated |
