# Supplementary material for: Low expression of long noncoding RNA CTC‐297N7.9 predicts poor prognosis in patients with hepatocellular carcinoma
Source: Cancer Med. 2019 Nov 1;8(18):7679–92. doi: 10.1002/cam4.2618 (PMC6912069; doi:10.1002/cam4.2618)
Supplement: Supplementary file 5 [file CAM4-8-7679-s005.docx]

**Table S2.** ROC analysis of the top 50 up-regulated and down-regulated DElncRNAs in HCC

| **lncRNA** | **AUC** | **P value** |
| --- | --- | --- |
| AFAP1-AS1 | 0.827 | <0.001 |
| AC079466.1 | 0.714 | <0.001 |
| HAGLR | 0.975 | <0.001 |
| RP11-556E13.1 | 0.858 | <0.001 |
| ST8SIA6-AS1 | 0.782 | <0.001 |
| LINC00176 | 0.944 | <0.001 |
| AC018890.6 | 0.746 | <0.001 |
| RP11-385J1.2 | 0.841 | <0.001 |
| RP11-242J7.1 | 0.818 | <0.001 |
| AC009014.3 | 0.653 | <0.001 |
| RP13-143G15.4 | 0.796 | <0.001 |
| RP11-431J24.2 | 0.779 | <0.001 |
| ZFPM2-AS1 | 0.758 | <0.001 |
| LUCAT1 | 0.756 | <0.001 |
| CECR7 | 0.688 | <0.001 |
| RP5-1120P11.1 | 0.798 | <0.001 |
| RP11-284F21.9 | 0.860 | <0.001 |
| RP11-284F21.10 | 0.878 | <0.001 |
| RP11-492E3.2 | 0.733 | <0.001 |
| CDKN2B-AS1 | 0.954 | <0.001 |
| LINC00488 | 0.613 | 0.009 |
| LINC01139 | 0.584 | 0.053 |
| LINC00689 | 0.570 | 0.107 |
| RP11-30J20.1 | 0.605 | 0.016 |
| LINC00511 | 0.808 | <0.001 |
| LINC01436 | 0.702 | <0.001 |
| CRNDE | 0.842 | <0.001 |
| RP11-284F21.7 | 0.861 | <0.001 |
| CASC9 | 0.617 | 0.007 |
| RP4-565E6.1 | 0.800 | <0.001 |
| LINC01451 | 0.859 | <0.001 |
| TEX41 | 0.797 | <0.001 |
| LINC01116 | 0.890 | <0.001 |
| DUXAP8 | 0.810 | <0.001 |
| RP11-20D14.6 | 0.671 | <0.001 |
| ELFN1-AS1 | 0.635 | 0.002 |
| RP11-785D18.3 | 0.528 | 0.519 |
| RP3-323A16.1 | 0.797 | <0.001 |
| RP11-476K15.1 | 0.662 | <0.001 |
| RP11-401P9.4 | 0.646 | 0.001 |
| RP11-150O12.3 | 0.700 | <0.001 |
| LINC00494 | 0.526 | 0.557 |
| FLJ44511 | 0.865 | <0.001 |
| C21orf37 | 0.667 | <0.001 |
| RP11-838N2.4 | 0.839 | <0.001 |
| RP11-567G11.1 | 0.629 | 0.003 |
| C17orf82 | 0.961 | <0.001 |
| CTD-2015G9.2 | 0.647 | 0.001 |
| LINC01426 | 0.807 | <0.001 |
| MAFG-AS1 | 0.953 | <0.001 |
| CTC-505O3.2 | 0.823 | <0.001 |
| RP11-767I20.1 | 0.830 | <0.001 |
| RP11-273G15.2 | 0.825 | <0.001 |
| AC010969.1 | 0.725 | <0.001 |
| FLJ22763 | 0.815 | <0.001 |
| RP11-96D1.6 | 0.830 | <0.001 |
| RP11-1080G15.1 | 0.690 | <0.001 |
| AC012613.2 | 0.625 | 0.004 |
| RP11-179K3.2 | 0.801 | <0.001 |
| LINC00885 | 0.820 | <0.001 |
| AP003774.6 | 0.746 | <0.001 |
| RP11-772C9.1 | 0.801 | <0.001 |
| FAM99A | 0.815 | <0.001 |
| RP11-701P16.5 | 0.714 | <0.001 |
| WARS2-IT1 | 0.839 | <0.001 |
| RP11-31F19.1 | 0.779 | <0.001 |
| HHIP-AS1 | 0.890 | <0.001 |
| AL161668.5 | 0.842 | <0.001 |
| CLRN1-AS1 | 0.691 | <0.001 |
| RP11-109A6.2 | 0.724 | <0.001 |
| RP11-328K4.1 | 0.855 | <0.001 |
| TMEM26-AS1 | 0.710 | <0.001 |
| CTB-61M7.2 | 0.763 | <0.001 |
| CTC-526N19.1 | 0.895 | <0.001 |
| LINC01430 | 0.869 | <0.001 |
| LINC00238 | 0.865 | <0.001 |
| KBTBD11-OT1 | 0.841 | <0.001 |
| RP1-232P20.1 | 0.849 | <0.001 |
| RP11-205M3.3 | 0.848 | <0.001 |
| RP11-6B4.1 | 0.880 | <0.001 |
| HAND2-AS1 | 0.910 | <0.001 |
| RP11-422N16.3 | 0.805 | <0.001 |
| HAO2-IT1 | 0.845 | <0.001 |
| AC004540.4 | 0.893 | <0.001 |
| RP11-863K10.7 | 0.918 | <0.001 |
| RP11-830F9.5 | 0.889 | <0.001 |
| AC004538.3 | 0.902 | <0.001 |
| RP11-252E2.2 | 0.876 | <0.001 |
| RP11-295M3.4 | 0.902 | <0.001 |
| AF131217.1 | 0.889 | <0.001 |
| AC104809.2 | 0.864 | <0.001 |
| RP11-676J12.7 | 0.830 | <0.001 |
| RP11-598F7.3 | 0.820 | <0.001 |
| CTC-537E7.3 | 0.904 | <0.001 |
| FENDRR | 0.924 | <0.001 |
| CTC-297N7.9 | 0.856 | <0.001 |
| AP000439.1 | 0.866 | <0.001 |
| LINC00907 | 0.713 | <0.001 |
| AC016999.2 | 0.780 | <0.001 |
| LINC01093 | 0.952 | <0.001 |

*Abbreviations: AUC = area under the curve.*
