# Supplementary material for: Low expression of long noncoding RNA CTC‐297N7.9 predicts poor prognosis in patients with hepatocellular carcinoma
Source: Cancer Med. 2019 Nov 1;8(18):7679–92. doi: 10.1002/cam4.2618 (PMC6912069; doi:10.1002/cam4.2618)
Supplement: Supplementary file 6 [file CAM4-8-7679-s006.docx]

**Table S3.** Univariate survival analysis of the DElncRNAs with AUC>0.85

| **lncRNA** | **OS** | | |  | **DFS** | | |
| --- | --- | --- | --- | --- | --- | --- | --- |
|  | Coef | HR | P value |  | Coef | HR | P value |
| HAGLR | 0.007 | 1.007 | 0.418 |  | 0.010 | 1.010 | 0.162 |
| RP11-556E13.1 | 0.009 | 1.009 | 0.727 |  | -0.001 | 0.999 | 0.732 |
| LINC00176 | 0.015 | 1.015 | 0.185 |  | 0.009 | 1.009 | 0.488 |
| RP11-284F21.9 | 0.022 | 1.022 | 0.134 |  | 0.030 | 1.030 | 0.032 |
| RP11-284F21.10 | 0.014 | 1.014 | 0.012 |  | 0.008 | 1.008 | 0.132 |
| CDKN2B-AS1 | 0.230 | 1.259 | 0.055 |  | 0.302 | 1.353 | 0.002 |
| RP11-284F21.7 | 0.026 | 1.027 | 0.010 |  | 0.010 | 1.010 | 0.313 |
| LINC01451 | -0.008 | 0.992 | 0.519 |  | 0.002 | 1.002 | 0.877 |
| LINC01116 | 0.015 | 1.016 | 0.189 |  | 0.011 | 1.011 | 0.295 |
| FLJ44511 | 0.058 | 1.059 | 0.265 |  | 0.022 | 1.022 | 0.635 |
| C17orf82 | 0.014 | 1.015 | 0.837 |  | 0.089 | 1.093 | 0.096 |
| MAFG-AS1 | 0.029 | 1.030 | 0.001 |  | 0.015 | 1.015 | 0.139 |
| HHIP-AS1 | 0.006 | 1.006 | 0.567 |  | -0.012 | 0.988 | 0.537 |
| RP11-328K4.1 | -0.014 | 0.986 | 0.053 |  | -0.001 | 0.999 | 0.914 |
| CTC-526N19.1 | -0.002 | 0.998 | 0.975 |  | 0.034 | 1.035 | 0.259 |
| LINC01430 | -0.061 | 0.941 | 0.324 |  | 0.005 | 1.005 | 0.922 |
| LINC00238 | -0.078 | 0.925 | 0.130 |  | -0.032 | 0.968 | 0.342 |
| RP11-6B4.1 | 0.005 | 1.005 | 0.430 |  | -0.002 | 0.998 | 0.744 |
| HAND2-AS1 | -0.143 | 0.867 | 0.444 |  | -0.231 | 0.794 | 0.210 |
| AC004540.4 | -0.019 | 0.981 | 0.554 |  | 0.003 | 1.003 | 0.920 |
| RP11-863K10.7 | -0.213 | 0.808 | 0.097 |  | -0.128 | 0.880 | 0.213 |
| RP11-830F9.5 | -0.112 | 0.894 | 0.192 |  | -0.006 | 0.994 | 0.925 |
| AC004538.3 | -0.226 | 0.797 | 0.218 |  | -0.314 | 0.730 | 0.066 |
| RP11-252E2.2 | -0.058 | 0.943 | 0.168 |  | -0.041 | 0.960 | 0.212 |
| RP11-295M3.4 | -0.017 | 0.983 | 0.780 |  | 0.015 | 1.015 | 0.754 |
| AF131217.1 | -0.427 | 0.652 | 0.640 |  | -0.514 | 0.598 | 0.516 |
| AC104809.2 | 0.000 | 1.000 | 0.988 |  | -0.003 | 0.997 | 0.775 |
| CTC-537E7.3 | -0.047 | 0.954 | 0.085 |  | -0.005 | 0.995 | 0.760 |
| FENDRR | -0.694 | 0.499 | 0.344 |  | -0.298 | 0.742 | 0.548 |
| CTC-297N7.9 | -0.139 | 0.870 | 0.000 |  | -0.063 | 0.939 | 0.013 |
| AP000439.1 | -0.011 | 0.989 | 0.734 |  | -0.013 | 0.987 | 0.694 |
| LINC01093 | -0.001 | 0.999 | 0.654 |  | -0.001 | 0.999 | 0.770 |

*Abbreviations: OS = overall survival; DFS = disease-free survival; Coef = coefficient; HR = hazard ratio; 95% CI = 95% confidence interval.*
