# Supplementary material for: Low expression of long noncoding RNA CTC‐297N7.9 predicts poor prognosis in patients with hepatocellular carcinoma
Source: Cancer Med. 2019 Nov 1;8(18):7679–92. doi: 10.1002/cam4.2618 (PMC6912069; doi:10.1002/cam4.2618)
Supplement: Supplementary file 7 [file CAM4-8-7679-s007.docx]

**Table S4.** Univariate regression analysis of clinicopathological characteristics for OS and DFS in patients from the TCGA

| Clinicopathological Characteristics | | OS | | |  | DFS | | |
| --- | --- | --- | --- | --- | --- | --- | --- | --- |
|  |  | Coef | HR (95% CI) | P value^b^ |  | Coef | HR (95% CI) | P value^b^ |
| Age(years) | >60 VS. ≤60 | 0.193 | 1.213 (0.854 - 1.723) | 0.281 |  | -0.036 | 0.964 (0.715 - 1.300) | 0.812 |
| Gender | Male VS. Female | -0.184 | 0.832 (0.582 - 1.191) | 0.316 |  | -0.039 | 0.962 (0.700 - 1.322) | 0.811 |
| Child-Pugh | B/C VS. A | 0.319 | 1.376 (0.788 - 2.402) | 0.261 |  | 0.201 | 1.222 (0.729 - 2.049) | 0.446 |
| Hepatitis virus infection | Yes VS. No | -0.685 | 0.504 (0.348 - 0.730) | <0.001^*^ |  | -0.188 | 0.829 (0.614 - 1.119) | 0.220 |
| Alcoholic liver | Yes VS. No | 0.038 | 1.039 (0.714 - 1.511) | 0.842 |  | 0.007 | 1.007 (0.733 - 1.383) | 0.966 |
| Cirrhosis | Yes VS. No | -0.111 | 0.895 (0.617 - 1.297) | 0.558 |  | 0.058 | 1.059 (0.777 - 1.444) | 0.715 |
| AFP(μg/L) | >400 VS. ≤400 | 0.183 | 1.201 (0.812 - 1.777) | 0.359 |  | 0.102 | 1.108 (0.776 - 1.580) | 0.573 |
| TNM stage | III/IV VS. I/II | 0.897 | 2.452 (1.721 - 3.495) | <0.001^*^ |  | 0.777 | 2.175 (1.584 - 2.986) | <0.001^*^ |
| Differentiation | Low VS. High/Moderate | 0.394 | 1.482 (1.044 - 2.106) | 0.028^*^ |  | 0.312 | 1.366 (1.007 - 1.853) | 0.045^*^ |
| Vascular invasion | Yes VS. No | 0.408 | 1.504 (1.058 – 2.137) | 0.023^*^ |  | 0.537 | 1.711 (1.266 – 2.312) | <0.001^*^ |
| CTC-297N7.9 Expression | High VS. Low^a^ | -0.783 | 0.457 (0.319 - 0.655) | <0.001^*^ |  | -0.441 | 0.643 (0.476 - 0.869) | 0.004^*^ |

*Abbreviations: Coef = regression coefficient; HR = hazard ratio; 95% CI = 95% confidence interval; AFP = alpha-fetoprotein.*

*a: The median expression level of CTC-297N7.9 was used as the cutoff. Low CTC-297N7.9 expression among the 184 patients was defined as a value below the 50th percentile; while high CTC-297N7.9 expression among the 184 patients was defined as a value above the 50th percentile.*

*b: Cox regression, *P<0.05.*
