# Supplementary material for: Low expression of long noncoding RNA CTC‐297N7.9 predicts poor prognosis in patients with hepatocellular carcinoma
Source: Cancer Med. 2019 Nov 1;8(18):7679–92. doi: 10.1002/cam4.2618 (PMC6912069; doi:10.1002/cam4.2618)
Supplement: Supplementary file 8 [file CAM4-8-7679-s008.docx]

**Table S5.** Multivariate regression analysis of clinicopathological characteristics for OS and DFS in patients from the TCGA

| Clinicopathological Characteristics | | OS | | |  | DFS | | |
| --- | --- | --- | --- | --- | --- | --- | --- | --- |
|  |  | Coef | HR (95% CI) | P value^b^ |  | Coef | HR (95% CI) | P value^b^ |
| Hepatitis virus infection | Yes VS. No | -0.582 | 0.559 (0.379 - 0.824) | 0.003^*^ |  | / | / | / |
| TNM stage | III/IV VS. I/II | 0.648 | 1.911 (1.318 - 2.772) | 0.001^*^ |  | 0.629 | 1.876 (1.346 - 2.615) | <0.001^*^ |
| Vascular invasion | Yes VS. No | / | / | / |  | 0.345 | 1.411 (1.030 - 1.934) | 0.032^*^ |
| CTC-297N7.9 Expression | High VS. Low^a^ | -0.782 | 0.457 (0.318 - 0.659) | <0.001^*^ |  | -0.333 | 0.717 (0.529 - 0.972) | 0.032^*^ |

*Abbreviations: Coef = regression coefficient; HR = hazard ratio; 95% CI = 95% confidence interval.*

*a: The median expression level of CTC-297N7.9 was used as the cutoff. Low CTC-297N7.9 expression among the 184 patients was defined as a value below the 50th percentile; while high CTC-297N7.9 expression among the 184 patients was defined as a value above the 50th percentile.*

*b: Cox regression, *P<0.05.*
